# Pharmacist-Led Collaborative Medication Management for the Elderly with Chronic Kidney Disease and Polypharmacy

**DOI:** 10.3390/ijerph18084370

**Published:** 2021-04-20

**Authors:** A Jeong Kim, Hayeon Lee, Eun-Jeong Shin, Eun-Jung Cho, Yoon Sook Cho, Hajeong Lee, Ju-Yeun Lee

**Affiliations:** 1 Department of Pharmacy, Seoul National University Hospital, Seoul 03080, Korea; anemone@snuh.org (A.J.K.); 30560@snuh.org (H.L.); eunjeong.shin@sksh.ae (E.-J.S.); whitegold@snuh.org (E.-J.C.); joys99@snuh.org (Y.S.C.); 2 Division of Nephrology, Department of Internal Medicine, Seoul National University Hospital, Seoul 03080, Korea; mdhjlee@gmail.com; 3 College of Pharmacy and Research Institute of Pharmaceutical Sciences, Seoul National University, Seoul 08826, Korea

**Keywords:** geriatric, potentially inappropriate medications, polypharmacy, medication management service, chronic kidney disease

## Abstract

Inappropriate polypharmacy is likely in older adults with chronic kidney disease (CKD) owing to the considerable burden of comorbidities. We aimed to describe the impact of pharmacist-led geriatric medication management service (MMS) on the quality of medication use. This retrospective descriptive study included 95 patients who received geriatric MMS in an ambulatory care clinic in a single tertiary-care teaching hospital from May 2019 to December 2019. The average age of the patients was 74.9 ± 7.3 years; 40% of them had CKD Stage 4 or 5. Medication use quality was assessed in 87 patients. After providing MMS, the total number of medications and potentially inappropriate medications (PIMs) decreased from 13.5 ± 4.3 to 10.9 ± 3.8 and 1.6 ± 1.4 to 1.0 ± 1.2 (both *p* < 0.001), respectively. Furthermore, the number of patients who received three or more central nervous system-active drugs and strong anticholinergic drugs decreased. Among the 354 drug-related problems identified, “missing patient documentation” was the most common, followed by “adverse effect” and “drug not indicated.” The most frequent intervention was “therapy stopped”. In conclusion, polypharmacy and PIMs were prevalent in older adults with CKD; pharmacist-led geriatric MMS improved the quality of medication use in this population.

## 1. Introduction

Inappropriate polypharmacy, defined as the use of one or more “medications that have no clear evidence-based indication, or no longer indicated or those carrying a substantially higher risk of adverse effects”, presents increasing challenges, especially in older adults [1,2]. Various strategies have been attempted to manage inappropriate polypharmacy [3]. A medication review and medication management service (MMS), aimed at improving the appropriateness of medication use, reducing medication-related harm, and improving clinical outcomes, is recommended by many guidance documents, and their implementation is increasing worldwide to address inappropriate polypharmacy [1,2,3]. A systematic review of strategic guidance for addressing inappropriate polypharmacy showed that most guidance paid attention to the need for regular medication review and some addressed the issue of non-adherence in older people [1]. Medication management via collaboration between pharmacists and physicians has been suggested as one of the most effective strategies for reducing medication-related clinical, social, and economic outcomes [4,5,6]. A collaborative care model with the participation of pharmacists showed significant improvement in medication related patient health outcome such as glycosylated hemoglobin A1c, cholesterol index and blood pressure as well as decreased hospitalization compared to that with usual care without the participation of pharmacists [4]. In addition, a recent meta-analysis showed that medication review by community pharmacists for the elderly with polypharmacy significantly reduced the risk of emergency department visits by 32% [7].

Quality of medication use—the degree to which medication use in individuals increases the likelihood of desired health outcomes [8]—has been used as one of the outcomes of medication management systems or pharmacist interventions. However, the items and measurement methods were different among studies [9,10,11].

Although the role of community pharmacy has extended to address inappropriate polypharmacy in community-dwelling older patients globally [12,13], MMS by non-dispensing pharmacists or pharmacist-led MMS for ambulatory older patients has not been implemented in Korea. Considering patient preference for hospital-level ambulatory care over clinic-level care in Korea [14] and the feasibility of collaboration between pharmacists and physicians within the same institution, implementing a pharmacist-led collaborative MMS in tertiary hospitals might be the first step towards extending pharmacist-led MMS.

Older adults with chronic kidney disease (CKD), in particular, are at high risk of polypharmacy because they typically have various comorbidities, such as diabetes, hypertension, cardiovascular disease, anemia, and bone and mineral disease [15]. Moreover, patients with renal insufficiency are especially vulnerable to drug-related adverse events, in part due to pharmacokinetic/pharmacodynamic changes as well as the use of multiple medications. Therefore, special precautions are required in terms of drug selection, drug interactions, and dose adjustment [16,17]. A pharmacist-led collaborative MMS for older adults visiting the nephrology clinic was established in a tertiary academic medical center in Korea with the goal of managing polypharmacy and improving the quality of care.

The aim of this study was to evaluate the impact of the newly implemented pharmacist-led collaborative geriatric MMS on the quality of medication use in older adults with polypharmacy visiting the nephrology clinic.

## 2. Materials and Methods

This single-center, retrospective, descriptive study was conducted at Seoul National University Hospital (SNUH). We evaluated the impact of geriatric MMS provided to patients visiting the nephrology clinic from May 2019 to December 2019 on the quality of medication use by retrospectively reviewing patient medical records.

SNUH is a 1700-bed tertiary care teaching hospital that provides medical services to more than 8900 outpatients daily as well as inpatient care. Outpatient geriatric MMS in SNUH began in 2018 in collaboration with specific practitioners and was targeted at geriatric patients who visited a specific department (rheumatology), with the ultimate goal of extending the service to all geriatric outpatients. After the service was well established for geriatric patients visiting the rheumatology department, it was expanded to geriatric patients visiting the nephrology clinic. This was because in a preliminary study, the proportion of older patients who visited the nephrology clinic ranked in the top three among the ambulatory elderly patients with excessive polypharmacy, and these patients were expected to have many drug-related problems (DRPs) due to the nature of kidney disease.

Before the start of the service, pharmacists and physicians, by consensus, established a protocol regarding patient selection, criteria for evaluating medication use, patient counseling, and referral and communication procedures. To provide standardized pharmaceutical care for elderly patients with polypharmacy, the pharmacy department developed a computerized pharmacist-led geriatric MMS support program that enables pharmacists to perform a structured geriatric medication review and document the services provided.

Under this program, designated pharmacists provide weekly patient counseling (one half-day session per week). They have full access to medical records, except for psychiatric counseling records. Before the session, pharmacists pre-screened and selected patients with scheduled appointments based on the number of prescribed medications, use of potentially inappropriate medications (PIMs) by the patient, and the number of medical departments visited by the patient. Thereafter, pharmacists obtained primary medical and medication histories and pre-evaluated the appropriateness of the prescribed medications before counseling.

During patient counseling, pharmacists confirmed and updated medication lists to include substances, such as medicines prescribed outside SNUH, self-medicated over-the-counter (OTC) medications, and dietary supplements. After confirming patient medication history, pharmacists evaluated drug-related issues, including medication adherence, effectiveness of medications, current or past adverse drug reactions, drug–drug interactions, patient dependency on medications that were not indicated at the time of counseling, and the necessity for deprescribing. Finally, pharmacists counseled patients regarding general precautions to be taken during medication use and empowered the patients to ask their prescribing physicians outside the institution about the possibility of deprescribing medications deemed inappropriate or unnecessary. Furthermore, they directly contacted the physician, if necessary. They also provided written recommendations to the collaborating physicians before and after patient counseling. Pharmaceutical interventions to resolve DRPs were performed through pharmacist-patient and pharmacist-physician interactions.

We retrieved the medical records of patients who visited the nephrology clinic and received geriatric MMS from May 2019 to December 2019. Further, we descriptively analyzed pharmacist intervention records and described the change in the quality of medication use during pharmacist-led MMS. Medication use after providing geriatric MMS was followed up until the end of March 2020. All substances used by the patients were classified using the Anatomical Therapeutics Chemical (ATC) Classification System of the World Health Organization; topical agents were excluded from the analysis.

The quality of medication use and inappropriate polypharmacy were measured based on the number of medications, number of PIMs, and proportion of patients on PIMs according to Beers criteria 2019 [18] and Screening Tool of Older Persons’ Prescriptions (STOPP)/Screening Tool to Alert to Right Treatment (START) criteria [19]. Concurrent use of 10 or more medications was defined as excessive polypharmacy. We also evaluated the number of central nervous system (CNS)-active drugs and strong anticholinergic agents, according to Beers criteria 2019 [18], and anticholinergic burden according to the Korean Anticholinergic Burden Scale (KABS) [20]. Pharmacist interventions were evaluated based on the frequencies and types of DRPs and related interventions using the PharmDISC classification tool [21].

Descriptive data are presented as percentages or mean values with standard deviations. Comparisons of the quality of medication use before and after intervention were performed with a paired *t*-test or the Wilcoxon signed-rank test for continuous variables and the McNemar and McNemar-Bowker test of symmetry for categorical variables. All data were considered significant if the *p*-value was less than 0.05. Analyses were performed using the SAS software package version 9.3 (SAS Institute, Inc., Cary, NC, USA).

## 3. Results

### 3.1. Patient Characteristics

A total of 95 patients who received collaborative geriatric MMS were included in the analysis. More than two counseling sessions were provided to 43.2% of the patients during the study period. The average age of the patients was 74.9 ± 7.3 years, and 58.9% were male patients. According to the Modification of Diet in Renal Disease estimated glomerular filtration rate (MDRD eGFR), 43.2%, 16.8%, and 23.2% of the patients had stage 3, 4, and 5 CKD, respectively. Approximately, 17.9% of the patients were on hemodialysis, and 76.5% of them attended out-of-hospital dialysis facilities. The most frequent comorbidities were hypertension (76.8%), diabetes (45.3%), genitourinary disease (27.4%), and ischemic heart disease (24.2%). These patients were included in the analysis of DRPs (Table 1).

### 3.2. Change in Quality of Medication Use during Geriatric MMS

Eight patients who were not followed up until March 2020 were excluded, and 87 patients were finally included for the analysis of change in quality of medication use after providing MMS. Their characteristics are presented in Table 1. The number of overall medications decreased from 13.5 ± 4.3 to 10.9 ± 3.8 (*p* < 0.001), and the proportion of patients with excessive polypharmacy reduced from 85.1% to 59.8% after geriatric MMS (*p* < 0.001). Further, the number of PIMs per patient decreased from 1.6 ± 1.4 to 1.0 ± 1.2 (*p* < 0.001). A reduction of at least one PIM was observed in 80.5% of the patients after providing geriatric MMS. The proportion of patients who used at least one PIM and three or more PIMs significantly decreased from 77.0% to 59.8% and 23.0% to 10.3%, respectively (*p* < 0.001). The number of patients who were taking three or more CNS-active drugs was 21 (24.1%) at baseline and decreased to 15 (17.2%) after receiving MMS (*p* = 0.01). The proportions of patients on any and two or more strong anticholinergic drugs reduced from 34.5% to 20.7% and 4.6% to 2.3%, respectively (*p* = 0.003). The anticholinergic burden score, determined using KABS, decreased from 2.7 ± 2.6 at baseline to 1.8 ± 2.2 after providing geriatric MMS (*p* < 0.001) (Table 2).

### 3.3. Types of DRPs and Pharmacist Interventions

Among the 95 patients who received geriatric MMS, 354 DRPs were identified in 94 patients. The most frequent type of DRP was “missing patient documentation” (82 cases in 69 patients), which included an unrecorded medication history of drugs prescribed outside the institution, OTC medications, and dietary supplements; followed by “adverse effect” (43 cases in 33 patients); “drug not indicated” (40 cases in 36 patients); “contraindication” (40 cases in 34 patients); and “insufficient compliance” (33 cases in 32 patients). The most frequent intervention type was “therapy stopped” (111 cases, 31.4%), followed by “clarification/addition of information” (82 cases, 23.2%), “in-depth counseling of patient” (46 cases, 13.0%), and “proposition of therapy monitoring” (42 cases, 11.9%). The overall acceptance rate for recommendations was 81.7%, and the rates for “therapy stopped”, “therapy started”, “dose adjustment”, and “substitution” were 73.8%, 91.7%, 79.2%, and 85.7%, respectively (Table 3).

More than 60% of the interventions were delivered only to patients. “Therapy stopped” was the most frequent (40.7%) among these interventions, followed by “in-depth counseling of patient” (21.5%) and “proposition of therapy monitoring” (19.6%) (Table S1).

## 4. Discussion

This study showed that a pharmacist-led collaborative geriatric MMS for older adults with polypharmacy visiting the nephrology clinic had a significant impact on improving the quality of medication use with regard to reducing excessive polypharmacy, PIM use, CNS-active drug use, and anticholinergic burden. These results were in line with previous findings that showed positive benefit of a collaborative care approach offering medication review by clinical pharmacists, wherein the quality of pharmacotherapy had improved [6]. Although developed in collaboration with physicians, the geriatric MMS model of this study, which was feasible in the ambulatory clinic setting of this tertiary hospital, might not be a fully collaborative model because identification of the target patients was led by a pharmacist rather than a physician’s referral [22].

As we targeted patients for providing this geriatric MMS and considered all medications including OTC drugs and nutritional supplements, the high prevalence of excessive polypharmacy and inappropriate polypharmacy was as expected and similar to that reported previously in patients with CKD [15]. Approximately 85% of the patients were taking 10 or more medications, and 77% of the patients were taking at least one PIM or had therapeutic duplications at baseline. However, the total number of medications and PIMs decreased significantly after providing geriatric MMS.

The acceptance rate of recommendations was 81.7%, which was higher than that reported in a community setting [12] and similar to that in a hospital setting for patients with CKD [16]. The most frequent DRP in this study was “missing patient documentation”, which in most cases consisted of missed medication history, and could be explained by the ambulatory clinical setting, wherein documenting the best possible medication history of a patient was impossible due to the short duration of consultations with physicians. Performing medication reconciliation (MR) in ambulatory care settings could increase the possibility of safe medication use despite the unknown clinical outcome [23,24].

The most prevalent pharmacist intervention in this study was drug discontinuation (31.4%). In some cases, this intervention was directly communicated to the physician, whereas in others, it was communicated through patients because prescribing physicians were out of the institution, and it was difficult to directly communicate with them. Unlike this study, some previous studies showed that only a small proportion of interventions by clinical pharmacists were related to drug discontinuation [6]. This difference might be explained by the difference in the patient population between the studies because we selected older patients receiving polypharmacy. In addition, as most of our patients had impaired renal function or were on dialysis, OTC drugs or dietary supplements needed to be discontinued. This type of intervention significantly reduced the number of medications used.

In this study, we evaluated the effectiveness of geriatric MMS for ambulatory older adults with CKD or at risk for CKD. While previous pharmacy practices focused on the management of CKD complications [16], geriatric MMS in this study focused on evaluation of use of PIMs in older adults for whom MR was performed, general precautions about PIMs, patient education regarding medication use, including the use of OTC drugs and dietary supplements, duplication of medications from visiting multiple physicians, and strategies to reduce inappropriate polypharmacy.

There are several important limitations of this study, which should be addressed. First, this study had no control group to determine the clinical outcomes of geriatric MMS owing to the retrospective nature of the study design. However, this study evaluated the benefit of collaborative geriatric MMS by comparing the quality of medication use among patients before and after providing geriatric MMS. Further, a retrospective study design has several other inherent limitations, such as attrition bias, selection bias, and missing data. Second, a certain degree of recall bias might have existed because we did not limit data gathering on medication use to medicines prescribed in our institution, and patients may have under-reported the medications taken. Third, all our patients were treated by a specialist nephrologist; therefore, our findings may not be generalizable to community-dwelling geriatric patients. Finally, the number of patients included in this analysis was small, and follow-up was not long enough to evaluate the long-term outcomes of geriatric MMS.

There is a global trend to involve pharmacists in MMS because of their specific medication-related knowledge. However, pharmacist-led geriatric MMS is neither a common practice in Korea nor covered by health insurance. The findings of this study offer new insights into the benefits of collaborative geriatric MMS, and this study is one of the first to investigate the impact of a geriatric MMS for ambulatory patients in Korea.

## 5. Conclusions

This study showed that polypharmacy and PIMs were prevalent in older adults visiting the nephrology clinic in a tertiary hospital. A newly implemented pharmacist-led collaborative geriatric MMS improved the quality of medication use in terms of reduction in the number of PIMs, chronic medications, CNS-active drugs, and anticholinergic burden in this population.

## Figures and Tables

**Table 1:** Baseline characteristics of patients who received geriatric medication management services.

Characteristics	DRP Analysis (N = 95) *n* (%)	Post-Intervention Analysis (N = 87) *n* (%)
Age, years, mean ± SD	74.9 ± 7.3	75.1 ± 7.3
65–74 years	48 (50.5)	43 (49.4)
75–84 years	36 (37.9)	33 (37.9)
≥85 years	11 (11.6)	11 (12.6)
Sex, male	56 (58.9)	51 (58.6)
Chronic kidney disease stages based on MDRD eGFR		
Stage 1 (≥90 mL/min/1.73 m^2^)	0 (0)	0 (0)
Stage 2 (60–89 mL/min/1.73 m^2^)	16 (16.8)	14 (16.1)
Stage 3 (30–59 mL/min/1.73 m^2^)	41 (43.2)	39 (44.8)
Stage 4 (15–29 mL/min/1.73 m^2^)	16 (16.8)	16 (18.4)
Stage 5 (<15 mL/min/1.73 m^2^)	22 (23.2)	18 (20.7)
Patients on hemodialysis	17 (17.9)	14 (16.1)
Co-morbid disease		
Hypertension	73 (76.8)	67 (77.0)
Diabetes mellitus	43 (45.3)	39 (44.8)
Genitourinary disease	26 (27.4)	25 (28.7)
Ischemic heart disease	23 (24.2)	21 (24.1)
Depression or other psychiatric disease	10 (10.5)	10 (11.5)
Dementia	10 (10.5)	9 (10.3)
Atrial fibrillation	8 (8.4)	8 (9.2)
Heart failure	6 (6.3)	6 (6.9)

DRP, drug-related problem; MDRD eGFR, modification of diet in renal disease estimated glomerular filtration rate.

**Table 2:** Changes in quality of medication use in patients undergoing geriatric medication management service (MMS) (N = 87).

	Pre-MMS *n* (%)	Post-MMS *n* (%)	*p*-Value
Number of medications, mean ± SD			
Medications including OTCs, dietary supplements *	13.5 ± 4.3	10.9 ± 3.8	<0.001
Self-medications including OTCs, dietary supplements	1.1 ± 1.3	0.6 ± 1.1	<0.001
Prescription drugs received outside institution	1.9 ± 3.4	1.4 ± 2.9	<0.001
Excessive polypharmacy	74 (85.1)	52 (59.8)	<0.001
Number of potentially inappropriate medications, mean ± SD	1.6 ± 1.4	1.0 ± 1.2	<0.001
0	20 (23.0)	35 (40.2)	<0.001
1–2	47 (54.0)	43 (49.4)	
3 or more	20 (23.0)	9 (10.3)	
Presence of duplicated medications	12 (13.8)	5 (5.7)	0.008
Number of CNS active drugs			
0	33 (37.9)	40 (46.0)	0.01
1–2	33 (37.9)	32 (36.8)	
3 or more	21 (24.1)	15 (17.2)	
Number of strong anticholinergics			
0	57 (65.5)	69 (79.3)	0.003
1	26 (29.9)	16 (18.4)	
2 or more	4 (4.6)	2 (2.3)	
Average KABS score, mean ± SD	2.7 ± 2.6	1.8 ± 2.2	<0.001
KABS score ≥ 3	40 (46.0)	28 (32.2)	

* excluding topical agents; CNS, central nervous system; KABS, Korean Anticholinergic Burden Scale; OTC, over-the-counter medications; MMS, medication management service.

**Table 3:** Types of baseline drug-related problems and pharmacist interventions.

Types of Drug Related Problems	Cases	Patients
C1.7 Missing patient documentation (medication history) ^a^	82 (23.2%)	69 (72.6%)
C1.6 Adverse effect	43 (12.1%)	33 (34.7%)
C1.4 Drug not indicated	40 (11.3%)	36 (37.9%)
C1.2 Contraindication	40 (11.3%)	34 (35.8%)
C5.1 Insufficient compliance	33 (9.3%)	32 (33.7%)
C1.5 Duplication	27 (7.6%)	20 (21.1%)
C1.1 No concordance with guidelines, only suboptimal therapy possible	23 (6.5%)	20 (21.1%)
C5.3 Concerns about the treatment	14 (4.0%)	12 (12.6%)
C3.3 Inappropriate monitoring	11 (3.1%)	11 (11.6%)
C3.2 Overdose	10 (2.8%)	10 (10.5%)
C3.4 Dose not adjusted to organ function	10 (2.8%)	9 (9.5%)
C1.3 Interaction	10 (2.8%)	8 (8.4%)
C4.1 Inappropriate timing or frequency of admin	9 (2.5%)	9 (9.5%)
C2.1 Inappropriate dosage form/administration route	1 (0.3%)	1 (1.1%)
C5.2 Insufficient knowledge	1 (0.3%)	1 (1.1%)
Intervention type	Case	Accepted
D5 Therapy stopped	111 (31.4%)	73.8% *
D10 Clarification/addition of information	82 (23.2%)	-
D7 In-depth counseling of patient (e.g., on adherence)	46 (13.0%)	-
D12 Proposition of therapy monitoring	42 (11.9%)	-
D6 Therapy started	24 (6.8%)	91.7%
D2 Dose adjustment	24 (6.8%)	79.2%
D4 Optimization of administration/route	9 (2.5%)	-
D11 Transmission of information	9 (2.5%)	-
D1 Substitution	7 (2.0%)	85.7%

* 76/103 because the data of eight cases were not available at the end of the study period. ^a^ includes update on medication history of drugs prescribed outside the institution, over-the-counter (OTC) medications, and dietary supplements.

## Data Availability

The datasets generated and/or analyzed during the present study are available from the corresponding author on reasonable request.

## Supplementary Materials

The following are available online at https://www.mdpi.com/article/10.3390/ijerph18084370/s1, Table S1: Types of interventions and drug-related problems depending on the target.
