# Supplementary material for: Pharmacist-Led Collaborative Medication Management for the Elderly with Chronic Kidney Disease and Polypharmacy
Source: Int J Environ Res Public Health. 2021 Apr 20;18(8):4370. doi: 10.3390/ijerph18084370 (PMC8074256; doi:10.3390/ijerph18084370)
Supplement: Supplementary file 1 [file ijerph-18-04370-s001.zip › ijerph-1144894-supplementary.pdf]

Supplementary Table.

Type of interventions and drug related problems depending on the target

**Interventions delivered to patients**	**214 (60.5%)**
**D5 Therapy stopped**	**87 (40.7%)**
C1.2 Contraindication	33
C1.4 Drug not indicated	28
C1.5 Duplication	17
C1.6 Adverse effect	8
C3.4 Dose not adjusted to organ function	1
**D7 In-depth counselling of patient (e.g., on adherence)**	**46 (21.5%)**
C5.1 Insufficient compliance	28
C5.3 Concerns about the treatment	11
C4.1 Inappropriate timing or frequency of admin	6
C5.2 Insufficient knowledge	1
**D12 Proposition of therapy monitoring**	**42 (19.6%)**
C1.6 Adverse effect	25
C3.3 Inappropriate monitoring	6
C1.3 Interaction	4
C1.5 Duplication	3
C5.3 Concerns about the treatment	2
C1.2 Contraindication	1
C5.1 Insufficient compliance	1
**D6 Therapy started**	**12 (5.6%)**
C1.1 No concordance with guidelines, only suboptimal therapy possible	10
C1.6 Adverse effect	2
**D11 Transmission of information**	**9 (4.2%)**
C3.3 Inappropriate monitoring	5
C5.1 Insufficient compliance	4
**D4 Optimization of administration/route**	**9 (4.2%)**
C4.1 Inappropriate timing or frequency of admin	2
C5.3 Concerns about the treatment	1
C1.3 Interaction	6
**D2 Dose adjustment**	**8 (3.7%)**
C1.6 Adverse effect	3
C3.4 Dose not adjusted to organ function	2
C4.1 Inappropriate timing or frequency of admin	1
C3.2 Overdose	2
**D1 Substitution**	**1 (0.5%)**
C1.1 No concordance with guidelines, only suboptimal therapy possible	1
**Interventions delivered to physicians**	**140 (39.5%)**

**D10 Clarification / addition of information**	**82 (58.6%)**
C1.7 Missing patient documentation (medication history)*	82
**D5 Therapy stopped**	**24 (17.1%)**
C1.4 Drug not indicated	12
C1.5 Duplication	7
C1.6 Adverse effect	2
C1.2 Contraindication	2
C3.4 Dose not adjusted to organ function	1
**D2 Dose adjustment**	**16 (11.4%)**
C1.6 adverse effect	2
C3.4 Dose not adjusted to organ function	6
C3.2 Overdose	8
**D6 Therapy started**	**12 (8.6%)**
C1.1 No concordance with guidelines, only suboptimal therapy possible	11
C1.6 adverse effect	1
**D1 Substitution**	**6 (4.3%)**
C1.2 Contraindication	4
C1.1 No concordance with guidelines, only suboptimal therapy possible	1
C2.1 Inappropriate dosage form/administration route	1
